# PA-MSHA induces apoptosis and suppresses metastasis by tumor associated macrophages in bladder cancer cells

**DOI:** 10.1186/s12935-017-0445-3

**Published:** 2017-08-17

**Authors:** Jianjun Liu, Xiaoyu Duan

**Affiliations:** Department of Urology, Henan Provincial People’s Hospital, No. 7 Weiwu Road, Zhengzhou, 450003 China

**Keywords:** Bladder, PA-MSHA, Macrophage, M1

## Abstract

**Background:**

The aim of the present study was to investigate effects of *Pseudomonas aeruginosa*-mannose-sensitive hemagglutinin (PA-MSHA) on the inhibition of the proliferation of bladder cancer cell lines and to further define its functional mechanisms.

**Methods:**

A rat model of bladder tumor was induced by intravesical N-methyl-N nitrosourea. The dynamic growth of tumor was measured by whole-body fluorescent imaging system. Morphological analysis was observed by hematoxylin–eosin staining and microscopic examination. The expression of Caspase 3 and E-Ca were detected by immunohistochemistry technique. Macrophages were separated by flow cytometry. The expression of cytokines was measured by qRT-PCR and western blot. Apoptosis ability was conducted by means of annexin V and propidium iodide. The abilities of invasion and migration were determined by transwell migration assay and scratch assay.

**Results:**

PA-MSHA and PA-MSHA + Fisetin groups inhibited the growth of tumor and increased the ratio of M1/M2. For one thing, PA-MSHA suppressed the invasive ability of the bladder tumor cell and promoted bladder tumor cell apoptosis. For another, it facilitated the expression of M1 cytokines and reduced expression of M2 cytokines. Furthermore, treated with PA-MSHA, mouse M1 phagocytosis rates were higher than that of M2 macrophages for bladder cancer lines.

**Conclusions:**

The data revealed that PA-MSHA might promote apoptosis and inhibit proliferation, invasion and migration of mouse bladder cancer cells by inducing M1 polarization.

## Background

Bladder cancer, one of the most common form of urologic cancers, is a primary clinical problem worldwide [1]. It is the main cause of death among urinary tumors in China [2]. The incidence of bladder cancer has been on the increase in recent years, with approximately 75–85% of the diagnosed tumors are non-muscle invasive bladder cancer [3]. The patients afflicted with non-muscle-invasive bladder cancer are susceptible to a high recurrence rate, ranging from 50 to 70% [4, 5]. Moreover, because of the aggressive nature of this cancer, 10–20% of non-muscle-invasive bladder cancer may rapidly progress to muscle-invasive disease [5, 6]. The prognosis of muscle-invasive bladder cancer is thus exceedingly poor, which is attributed to the high rate of metastasis [7].

Traditional treatments for bladder cancer were focused on the surgery. Complete transurethral resection with or without intravesical instillation is the standard treatment for bladder cancer. However, the greatest challenge in management is the potential for local recurrence [3, 4, 6]. Current treatment of immunotherapy, chemotherapy and radiotherapy have exhibited promising results and hence have been widely used as adjuvant therapies. Although these chemotherapeutic agents can achieve relatively acceptable effects, they are often insufferable on account of the strong systemic toxicity [8, 9]. Meanwhile, chemotherapy can cause plenty of side effects to the patients [10]. Furthermore, Angiogenesis inhibitors and monoclonal antibodies with anticancer effects cannot be used as systemic chemotherapeutic agents. Therefore, a novel adjuvant agent proven anticancer efficacy and minimal toxicity in patients with bladder cancer is urgently needed to improve overall survival.

*Pseudomonas* (P.) *aeruginosa* is a Gram-negative bacterium. The majority of human antibodies relevant to this microbe are produced by immunization with outer membrane proteins of *P. aeruginosa* [11, 12]. The PA-MSHA strain (a peritrichous *P. aeruginosa* strain with MSHA fimbriae) is established. It has been demonstrated to be effective in the improvement of immune response of patients with some types of cancer and other conditions including infection, trauma and chronic diseases [13–15]. PA-MSHA has also been reported to be a vaccine and widely used in anti-inflammatory, anti-infection, and even anti-cancer treatment. Furthermore, PA-MSHA possesses cytotoxicity because of the addition of MSHA, which has been reported to have anticarcinogenic ability against human gastric, breast cancer, hepatocarcinoma and nasopharyngeal cells [16–19]. It was verified that PA-MSHA could inhibit cell proliferation and prompt the apoptosis of gastric carcinoma cells by inducing M1 macrophage polarization [20]. M1 and M2 macrophages were found to be dynamic balance in glioma microenvironment and anti-CD47 treatment inhibited glioblastoma cell proliferation by promoting M1 macrophages polarization in vivo [21]. It was also reported that PA-MSHA could efficiently induce apoptosis and inhibit tumor proliferation, which was associated with inactivation of EGFR signaling pathway in bladder cancer [22]. These findings strongly suggest that PA-MSHA could promote bladder tumor cells apoptosis and inhibited tumor cells proliferation by inducing M1 macrophages polarization. However, potential effects of PA-MSHA to improve anticancer immunity in bladder cancer have not been understood yet.

Consequently, the objective of this study was to evaluate the influence of PA-MSHA on the inhibition of the proliferation of bladder cancer cell lines by mediating M1 and M2 polarization and to further elucidate the relevant functional mechanisms.

## Materials and methods

### Establishment of rat model of bladder cancer and experimental groups

This study was in accordance with the ethical standards and was approved by the Henan Provincial People’s Hospital. A total of 28 wistar rats weighing 200 ± 20 g and 7 weeks of age were used in this study. The mice were bred according to the Guidelines for the Care and Use of National Institutes of Health (NIH) at the Animal Center of Zhengzhou University. A rat model of bladder tumor was induced by intravesical MNU (Sigma, USA) reperfusion, once every 2 weeks for 10 weeks (2 mg/kg). Tumor-bearing rats were randomly divided into four groups (n = 7): the normal saline group (control), the fisetin treatment group (positive control) [23, 24], the PA-MSHA treatment group and the PA-MSHA + fisetin treatment group. Each group was given tail vein injection of saline, fisetin, PA-MSHA or combination of PA-MSHA and fisetin, twice per week every 3 days.

### Determination of bladder weight and expression of Caspase 3 and E-Ca

All rats were sacrificed in the 10th week after treatment, and then necropsy was performed. The total weight of the bladder was then determined as previously described [25]. Tumor tissues were fixed in 4% paraformaldehyde and embedded in paraffin. For morphological analysis, H&E staining and microscopic examination were performed on 3 µm-thick sections from paraffin-embedded tumor blocks. Immunohistochemical staining was probed with polyclonal anti-Caspase 3 antibody and polyclonal anti-E-cadherin antibody (Cell Signaling Technology, USA) according to the manufacturer’s instructions. Data was analyzed by Image-pro Plus Analysis system (Olympus, Japan). Macrophages were separated from tumor cells by their expression of CD11b and F4/80 for mouse macrophages and determined by flow cytometry [21].

### The role of PA-MSHA in the expression of M1 cytokines and M2 cytokines

To detect the role of PA-MSHA in murine bone marrow (BM)-derived macrophages, macrophages were separated and stimulated with different concentrations of PA-MSHA in vitro. To further explore whether PA-MSHA stimulation efficiency is time-dependent, murine BM-derived macrophages were isolated and cultured with 10^6^/mL PA-MSHA for 0, 6, 12, 24, 48, and 72 h. Then total RNA were extracted using TRIzol reagent (Invitrogen, USA), and cDNA were synthesized using one step PrimeScript miRNA cDNA Synthesis Kit (Takara Biotechnology, Dalian, China). The expression levels of cytokine (IL-12, TNF-α, IFN-γ, IL-4, IL-10 and TGF-β) were measured by qRT-PCR and the dates were determined by the 2^−ΔΔCT^ method [26]. All qRT-PCR reactions were performed three times. Subsequently, the protein expression levels of the cytokines were determined by Western blotting. In brief, cells in culture were lysed in RIPA buffer (Sigma, USA), then, the proteins were loaded onto 10% SDS-PAGE prior to being transferred onto nitrocellulose (NC) membranes (Amersham Bioscience, UK). Following blocking with skimmed milk, the membranes were incubated with the corresponding horseradish peroxidase (HRP)-conjugated secondary antibody at room temperature for 1 h. GAPDH was used as a control. Each protein sample was examined in triplicate.

### Apoptosis and invasion abilities assays in vitro

The mouse bladder cancer cell line BBN1617 cells or MB49 cells were incubated in the RPMI 1640 or culture supernatants of murine BM-derived macrophages treated with PA-MSHA. BBN1617 and MB49 were collected by low speed centrifugation (centrifuged at 2000 r/min for 3 min) and washed twice with ice-cold PBS. Then, 300 μL binding buffer were added to the cells. Afterward, the cells were stained with 5 μL propidium iodide (PI) and 5 μL annexin V at 4 °C for 5 min to 15 min in the dark. Apoptotic cells were counted by flow cytometry within 60 min using quadrant statistics of the annexin V-positive and propidium iodide-negative cells.

Cell invasion was determined by Transwell assays. The membrane of the chamber was coated with 30 mg/cm^2^ Matrigel (BD Biosciences, USA) for 1 h in 37 °C to form a matrix barrier. The lower chambers were filled with 600 μL of DMEM. The cells were suspended in DMEM to a concentration of 1 × 10^5^ cells per well and were loaded into each upper well with a volume of 200 µL. After the chambers cultured in 5% CO_2_ at 37 °C for 24 h, cells were fixed with methanol for 10 min and stained with crystal violet for 10 min. Subsequently, cells were washed with PBS. Counts were obtained from five random fields at 200× magnification.

Cell migration activity was analyzed using a scratch assay. BBN1617 and/or MB49 cells were plated onto 6-well plates at a density of 5 × 10^5^ cells per well and cultured to 100% confluence. Afterward, cells were scraped with a pipette 200 μL tip in a cross in the center of each well, washed with PBS, and immediately replaced with fresh low-serum medium. 48 h after growth, the migration distances of the cells were observed and photographed under a Nikon microscope (Minato) at a magnification of 200× for each group.

### Phagocytosis assay in vitro

To generate M1 or M2 macrophages, sorted BM cells were treated with recombinant mouse macrophage colony-stimulating factor (M-CSF). M1 polarization was achieved with further treatment through interferon gamma (IFN-γ) stimulation, followed by lipopolysaccharide (LPS). M2 polarization was obtained by further treatment with IL-4 and IL-13 [21].

Tumor cells were labeled with CFSE and co-incubated with murine BM-derived differentially polarized macrophages. Two different primary tumor cell lines (BBN1617 and MB49) were offered to either mouse M1 or M2 macrophages as targets. After the phagocytosis assays finished, staining of macrophages and the respective antibody master mix was done directly. The macrophage population was identified by macrophage markers CD11b and F4/80 targeted fluorescently labeled antibodies. Macrophages for each macrophage subtype was quantified by the percentage of CFSE+ events among CD206+ (mouse M2 macrophage), CD80+ (mouse M1 macrophages) events.

### Statistical analysis

All date were analyzed with SPSS17.0 (SPSS Inc, USA) and expressed as the mean ± S.D. Student’s t test was used to analyze differences between two groups. One-way ANOVA analysis was used to determine the multi-sample analysis. All statistical tests were two-sided, and P value less than 0.05 was considered significant.

## Results

### PA‑MSHA inhibited tumor growth in vivo

**Fig. 1:**
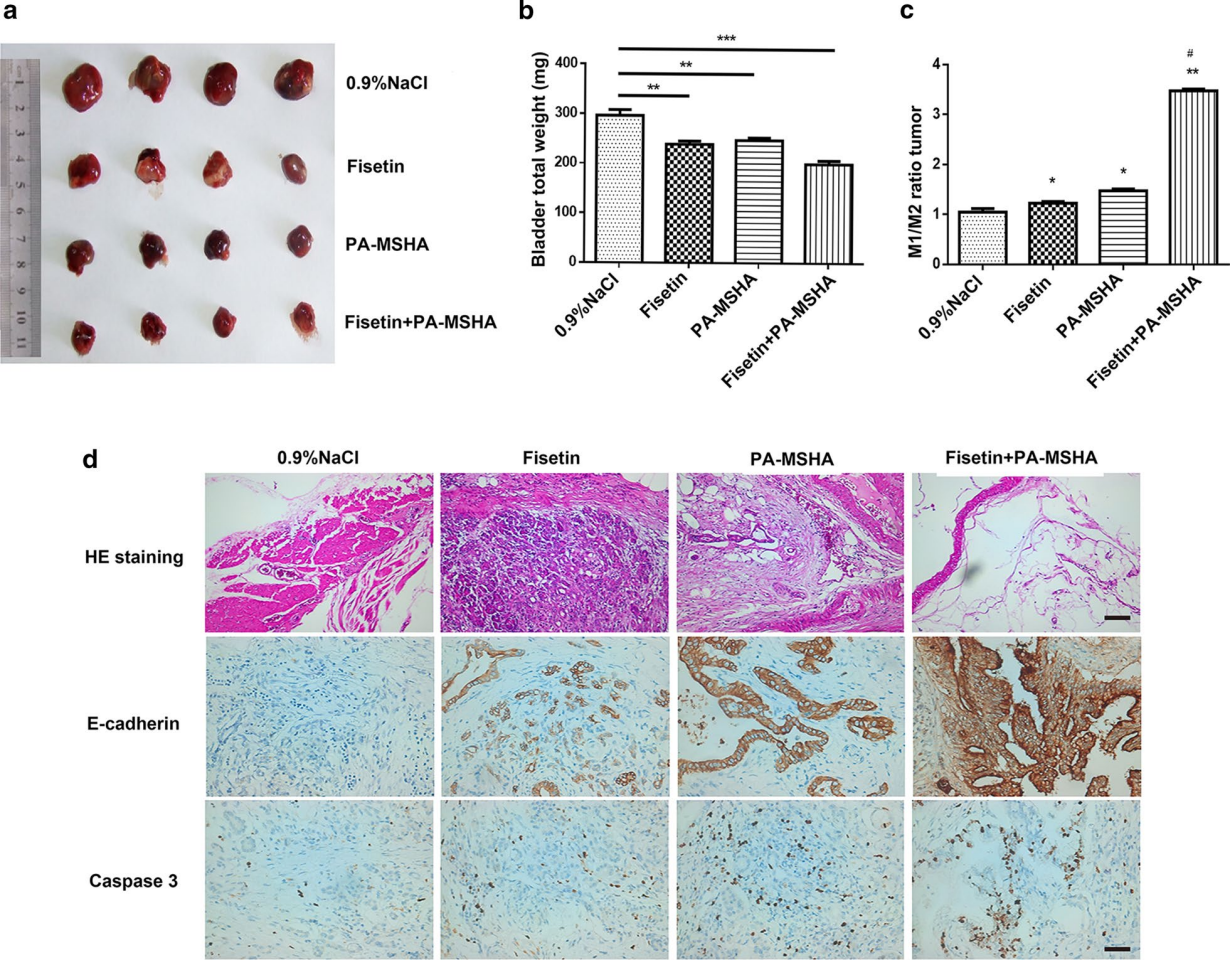
Effect of PA-MSHA, fisetin, PA-MSHA and fisetin in combination on tumor growth in vivo. **a** Representative images of tumors is shown. **b** Mice were killed, and tumor weight was measured. **c** Macrophages were separated from tumor cells and determined the M1/M2 ratio by flow cytometry. **d** Microphotographs of hematoxylin and eosin stained and tumor tissue sections. Immunohistochemical staining of Caspase 3 expression and E-Cadherin in rat bladder cancer. *Bar* 20 μm, *P < 0.05, **P < 0.01, ***P < 0.001, *versus 0.9% NaCl, ^#^versus PA-MSHA

In the first place, we investigated the effect of PA-MSHA on tumor which derived from a rat model of bladder tumor induced by intravesical MNU reperfusion. As shown in Fig. 1a, b, the average weight of the tumors from the PA-MSHA group as well as fisetin were significantly lower when compared to the 0.9% NaCl groups. The inhibition of tumor growth caused by PA-MSHA and fisetin in combination was much more markedly compared with those groups, indicating an inhibitory role of PA-MSHA on tumor growth in vivo and a significant enhancement of tumor growth inhibition combined with fisetin. As demonstrated in Fig. 1c, a dramatical increase in the ratio of M1/M2 was observed in PA-MSHA or PA-MSHA + Fisetin groups, suggesting the ability of PA-MSHA in inducing M1 polarization and crucial role of the increase of M1/M2 ratio in inhibiting tumor growth. Moreover, the results in Fig. 1d revealed that the tumor cells in the 0.9% NaCl groups exhibited diffuse patchy distribution, deeply stained nuclei and common mitotic figures while all of these disappeared gradually in the PA-MSHA or PA-MSHA + Fisetin groups. Meanwhile, a prominent higher expression of E-cadherin suggested that PA-MSHA could inhibit the invasive ability of the bladder tumor. Furthermore, the occurrence of brownish yellow in the cytoplasm reflected by the Caspase 3 positive staining demonstrated that PA-MSHA might hence promote bladder tumor cell apoptosis.

### PA-MSHA promoted the expression of M1 cytokines and reduced expression of M2 cytokines

**Fig. 2:**
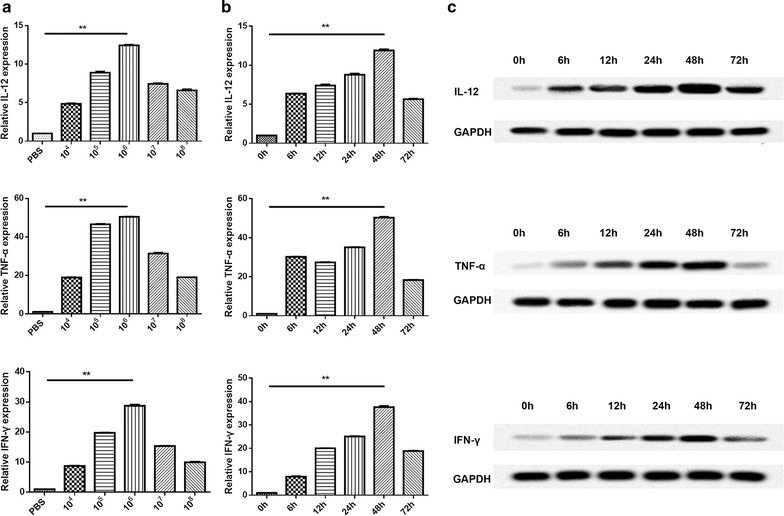
The expression levels of M1 cytokines from murine BM-derived macrophages stimulated by PA-MSHA. **a** The mRNA expression levels of IL-12, TNF-α and IFN-γ measured by qRT-PCR. **b** The mRNA expression levels of IL-12, TNF-α and IFN-γ from murine BM-derived macrophages treated with PA-MSHA (10^6^/mL) for 0, 6, 12, 24, 48, and 72 h. **c** The protein expression levels of IL-12, TNF-α and IFN-γ from MPMs treated with PA-MSHA (10^6^/mL) detected by Western blotting. *P < 0.05, **P < 0.01

**Fig. 3:**
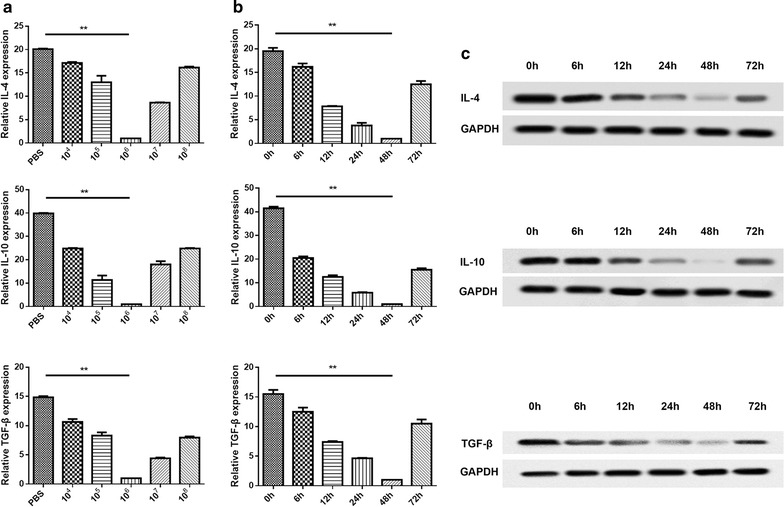
The expression levels of M1 cytokines from murine BM-derived macrophages stimulated by PA-MSHA. **a** The mRNA expression levels of IL-4, IL-10 and TGF-β measured by qRT-PCR. **b** The mRNA expression levels of IL-4, IL-10 and TGF-β from murine BM-derived macrophages treated with PA-MSHA (10^6^/mL) for 0, 6, 12, 24, 48, and 72 h. **c** The protein expression levels of IL-4, IL-10 and TGF-β from MPMs treated with PA-MSHA (10^6^/mL) detected by Western blotting. *P < 0.05, **P < 0.01

The role of PA-MSHA in murine BM-derived macrophage was investigated by qRT-PCR. The results shown in Figs. 2 and 3 indicated that the mRNA expression of IL-12, TNF-α and IFN-γ (the recognized M1 genes) were dramatically up-regulated (Fig. 2a) while IL-4, IL-10 and TGF-β (the recognized M2 genes) were significantly down-regulated (Fig. 3a) in different concentrations of PA-MSHA, especially in 10^6^/mL PA-MSHA.

To further verify whether PA-MSHA stimulation efficiency is time-dependent, qRT-PCR was performed. As shown in Figs. 2 and 3, the expression of IL-12, TNF-α and IFN-γ peaked at 48 h (Fig. 2b), while IL-4, IL-10 and TGF-β declined to a nadir at 48 h (Fig. 3b). These results revealed that the treatment time also played a critical role in activating the expression of M1 cytokines or inhibiting the expression of M2 cytokines.

In addition, the protein expression of these cytokines was verified by Western blotting and the results showed a similar trend with the qRT-PCR assay (Figs. 2c, 3c). In brief, these results suggested that PA-MSHA induced M1 macrophage polarization.

### PA-MSHA inhibited invasion and migration, promoted apoptosis of mouse bladder cancer cell in vitro

**Fig. 4:**
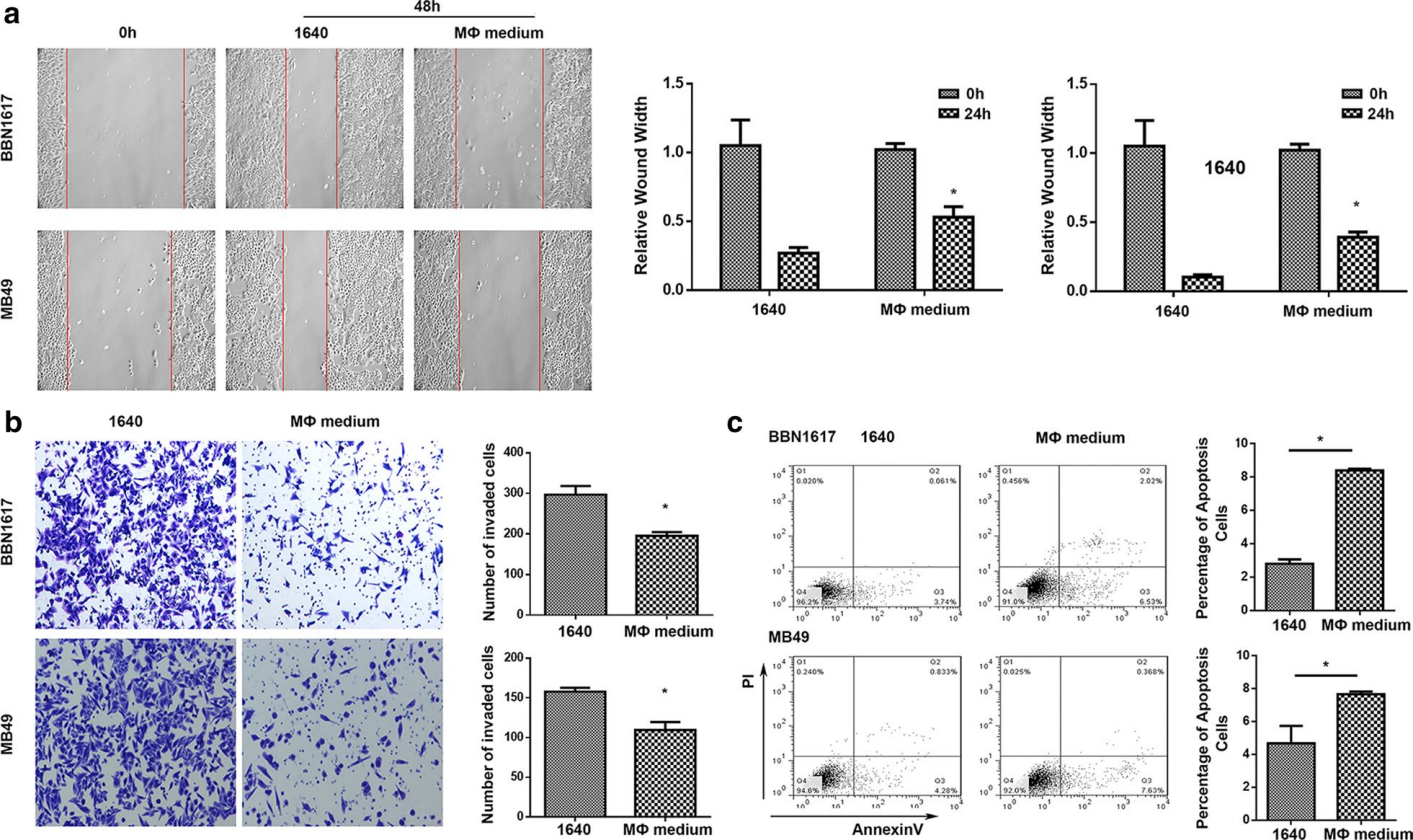
Effect of PA-MSHA on migration,invasion and apoptosis of BBN1617 and MB49 cells. **a** After scratching, BBN1617 and MB49 cells were incubated with PA-MSHA or RPMI 1640 for 48 h. Images were acquired at 0 and 48 h under the microscope. The *red lines* define the areas lacking migratory cells. A representative picture is shown here. **b** Comparison of invasion abilities. *Light images* of the Transwell invasion assay revealed that the number of invading UMUC-3 cells was significantly increased compared with 5637 cells and decreased compared with EJ cells. **c** Apoptosis of BBN1617 and MB49 cells were detected by flow cytometry. *Versus control group

After scratching, BBN1617 and MB49 cells were treated with PA-MSHA. The results demonstrated that PA-MSHA markedly inhibited cell migration in contrast to their respective control groups at 48 h (Fig. 4a). Furthermore, Fig. 4b showed that the number of invading BBN1617 or MB49 cells incubated with PA-MSHA decreased significantly compared with their respective control group. Therefore, light images of the transwell invasion assay revealed that PA-MSHA could inhibit invasion abilities of bladder cancer cells.

The apoptosis of mouse bladder cancer cells was explored with annexin V and PI staining, and the results were shown in Fig. 4c. It was shown that PA-MSHA could induce apoptosis of BBN1617 and MB49 cells. When accompanied with RPMI 1640, the rate of the apoptosis of BBN1617 and MB49 was enhanced, indicating the vital role of PA-MSHA in promoting apoptosis.

### Phagocytosis of M1 and M2 macrophages towards mouse bladder cancer cells in vitro

**Fig. 5:**
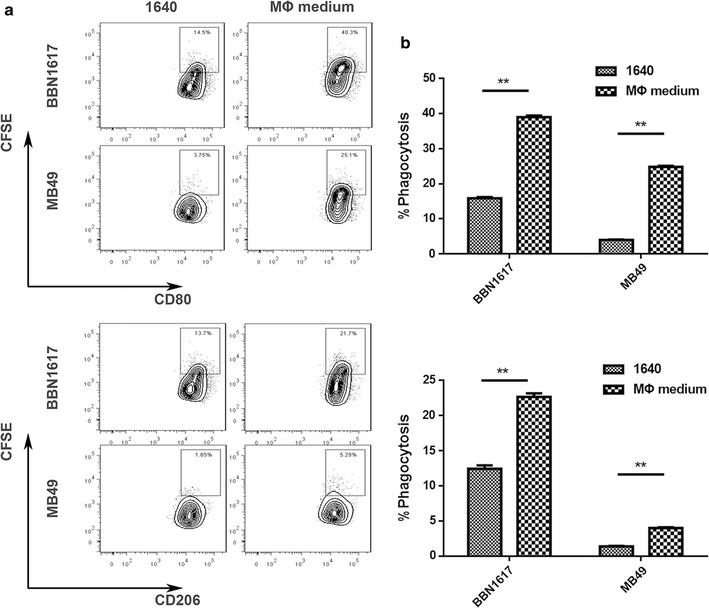
Differential phagocytosis rate of mouse M1 and M2 macrophages toward various mouse bladder cancer cells upon BBN1617 and MB49. **a** Representative flow cytometric phagocytosis assay of mouse M1 macrophages against CSFE-labeled BBN1617 and MB49 cells. **b** Representative flow cytometric phagocytosis assay of mouse M2 macrophages against CSFE-labeled BBN1617 and MB49 cells. **P < 0.01

The efficiency of phagocytosis of M1 or M2 macrophages towards mouse bladder cancer cells was analyzed ultimately. As shown in Fig. 5a, b, both M1 and M2 macrophages displayed significantly enhanced mean phagocytosis rates in contrast to controls in each individual cell line. Upon treatment of the PA-MSHA, mouse M1 phagocytosis rates were higher than in M2 macrophages for all lines.

## Discussion

Several recent findings have reported that PA-MSHA was effective in its anticancer cytotoxicity effect, such as nasopharyngeal cancer, hepatocarcinoma, gastric cancer and breast cancer cells [16, 18, 19, 27]. Besides, it was found that PA-MSHA could inhibit proliferation and induces apoptosis in human bladder cancer cell lines [28]. In this study, we attempted to assess the immunologic mechanism of PA-MSHA in bladder cancer. Our results indicated that PA-MSHA was able to inhibit tumor cell proliferation, invasion and migration as well as induce tumor cell apoptosis. Meanwhile, we found a dramatically increasing trend in the ratio of M1/M2 in this study, suggesting that PA-MSHA could induce M1 polarization and the increase of M1/M2 ratio might play a crucial role in inhibiting tumor growth. In addition, it has been reported that PA-MSHA treatment inhibited gastric carcinoma cells proliferation and migration by inducing polarization of M1 [20]. Thus, the following research primarily focused on the effect of PA-MSHA on macrophage polarization in bladder cancer.

Macrophages are originally produced from circulating monocytes. They differentiate within the tumor microenvironment and have a great impact on tumor progression and growth [29]. We separated murine BM-derived macrophages and treated them with PA-MSHA. The results showed that PA-MSHA treatment facilitated tumoricidal M1 polarization and up-regulated M1-related cytokines secretion, such as, IL-12, IFN-γ and TNF-α. Thus, a microenvironment which inhibited the proliferation of tumor cells and delayed tumor development was established. In addition, it was worth mentioning that low and middle doses of PA-MSHA (10^6^/mL) were more effective in activating M1-related cytokines and similar conditions was verified in gastric carcinoma [20]. However, higher doses of PA-MSHA slightly inhibited the expression of these cytokines compared with the treatment with 10^6^/mL PA-MSHA. Meanwhile, the duration of PA-MSHA treatment was also a vital factor in activating M1-related cytokines and the data showed that the peaks affected by PA-MSHA was at 48 h.

Moreover, elimination of tumor cells might occur through various mechanisms including phagocytic and non-phagocytic tumor cell killed by NK and neutrophils cells [30–32]. We hypothesized herein that M1 macrophages stimulated with PA-MSHA exhibited a larger phagocytic response towards mouse bladder cancer cells than M2 macrophages by PA-MSHA treatment in vitro.

Consequently, PA-MSHA induced M1 macrophages polarization and enhanced the phagocytosis of macrophage. Sequentially, it inhibited cell proliferation and migration and induced the apoptosis. It has been reported that the above effects on the polarization of M1 were associated with activation of NF-κB expression [33, 34]. Thereby, the signaling pathway of M1 macrophages polarization mediated by PA-MSHA was further investigated.

## Conclusions

The results of the current study suggested that PA-MSHA induced M1 polarization and therefore inhibited tumor growth and bladder cell proliferation. This mechanism might be a potential novel strategy for inhibiting bladder carcinoma progression in the mouse.

## Abbreviations

H&E: hematoxylin-eosin
IFN-γ: interferon gamma
LPS: lipopolysaccharide
M-CSF: macrophage colony-stimulating factor
MNU: N-methyl-N nitrosourea
PI: propidium iodide
PA-MSHA: *Pseudomonas aeruginosa*-mannose-sensitive hemagglutinin
